# Superflexible and Lead-Free Piezoelectric Nanogenerator as a Highly Sensitive Self-Powered Sensor for Human Motion Monitoring

**DOI:** 10.1007/s40820-021-00649-9

**Published:** 2021-04-30

**Authors:** Di Yu, Zhipeng Zheng, Jiadong Liu, Hongyuan Xiao, Geng Huangfu, Yiping Guo

**Affiliations:** State Key Laboratory of Metal Matrix Composites, School of Materials Science and Engineering, Shanghai Jiao Tong University, Shanghai, 200240 People’s Republic of China

**Keywords:** Superflexible, Piezoelectric sensors, Curie temperature, Human motion sensing

## Abstract

For traditional piezoelectric sensors based on poled ceramics, a low curie temperature (*T*_c_) is a fatal flaw due to the depolarization phenomenon. However, in this study, we find the low *T*_c_ would be a benefit for flexible piezoelectric sensors because small alterations of force trigger large changes in polarization. BaTi_0.88_Sn_0.12_O_3_ (BTS) with high piezoelectric coefficient and low *T*_c_ close to human body temperature is taken as an example for materials of this kind. Continuous piezoelectric BTS films were deposited on the flexible glass fiber fabrics (GFF), self-powered sensors based on the ultra-thin, superflexible, and polarization-free BTS-GFF/PVDF composite piezoelectric films are used for human motion sensing. In the low force region (1–9 N), the sensors have the outstanding performance with voltage sensitivity of 1.23 V N^−1^ and current sensitivity of 41.0 nA N^−1^. The BTS-GFF/PVDF sensors can be used to detect the tiny forces of falling water drops, finger joint motion, tiny surface deformation, and fatigue driving with high sensitivity. This work provides a new paradigm for the preparation of superflexible, highly sensitive and wearable self-powered piezoelectric sensors, and this kind of sensors will have a broad application prospect in the fields of medical rehabilitation, human motion monitoring, and intelligent robot.

**Supplementary Information:**

The online version contains supplementary material available at 10.1007/s40820-021-00649-9.

## Highlights

- Continuous piezoelectric BaTi_0.88_Sn_0.12_O_3_ (BTS) films were deposited on glass fiber fabrics successfully.
- Superflexible, highly sensitive self-powered piezoelectric sensors were fabricated based on polarization-free BTS.
- Low curie temperature would be a benefit for flexible piezoelectric sensors because small alterations of force will trigger large changes in polarization.
- The superflexible sensors are highly desirable for wearable devices to detect human motion.

**Supplementary Information:**

The online version contains supplementary material available at 10.1007/s40820-021-00649-9.

## Graphical abstract

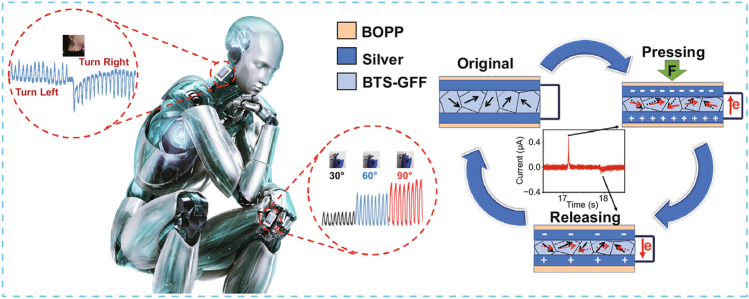

## Introduction

The detections of human body motions (i.e., movements of joints [1–3], diaphragmatic breathing [4], and heartbeats [5–7]) are increasingly interesting with the rapid development of biomedical field [8–10] and intelligent bionic robot industry [11–13]. Various kinds of sensors are required to be implanted in the human body, as a result, the recharge and overhaul of batteries are unavoidable. These tedious and invasive processes will cause harm to human health [14]. Self-powered sensors [15], aiming to address the challenge of power supply, have gained growing research interest in recent years [16–18]. Among effective energy harvesting technologies, piezoelectric nanogenerators [19–21] and triboelectric nanogenerators [22, 23] have been applied in body sensors network, smart insoles and many other aspects. The output of triboelectric nanogenerators depends on the coupling effect of contact electrification and electrostatic induction between two triboelectric layers [24]. It means that the triboelectric layer may be damaged in the process of multiple contact separation or sliding. Besides, the operating mechanism of contact mode nanogenerators may make the sealing and packaging of the devices more difficult, which limits the application of triboelectric nanogenerators in harsh environment [25]. Otherwise, the hard substrates or large size of high-output triboelectric nanogenerators will make the devices less wearable [26, 27]. Compared to triboelectric nanogenerators, piezoelectric nanogenerators have the merits of better portability and durability because piezoelectric materials are not easily damaged under the action of external forces, but their output performance is still lower than that of triboelectric nanogenerators [28–30]. Therefore, piezoelectric nanogenerators will have a better prospect of application in human motion sensing by improving the output performance.

Inorganic piezoelectric perovskite materials and piezoelectric polymers have been widely utilized in the fabrication of piezoelectric sensors. Piezoelectric polymers, such as poly(vinylidene fluoride) (PVDF) [31] and poly(vinylidene fluoride-trifluoroethylene) (P (VDF-TrFE)) [32], have the intrinsic flexibility and biocompatibility. However, the output performance of nanogenerators based on piezoelectric polymers are restricted by the low piezoelectric coefficient. Lead-based piezoelectric perovskite materials Pb(Zr,Ti)O_3_ and (1 − *x*) Pb(Mg,Nb)O_3_–*x*PbTiO_3_ have drawn much attentions due to the remarkable piezoelectric properties. However, it is well-known that lead is harmful for human health and ecological environment. Consequently, lead-free inorganic piezoelectric materials with outstanding performance have long been pursued. For instance, Ba_0.85_Ca_0.15_Zr_0.10_Ti_0.90_O_3_ [33], BiFeO_3_ [34] and (K, Na)NbO_3_-based [35–37] materials were reported to fabricate piezoelectric nanogenerators for energy harvesting and human motion sensing. Recently, it is found that stannum-doped BaTiO_3_ (BTS) ferroelectric ceramics have the highest piezoelectric coefficient (*d*_33_) in lead-free piezoceramics with a low Curie temperature (*T*_c_) of ~ 40 °C[38]. It is well known that the sensors based on hard ceramics will lose piezoelectricity due to the depolarization phenomenon once the work temperature is higher than *T*_c_. However, flexible piezoelectric nanogenerators can generate enough electric signals even without poling process and the low *T*_c_ would be a benefit for flexible sensors because a small alteration of force will trigger large changes in polarization [39]. Considering about the above facts, flexible BTS materials may have immense potential applications in wearable devices.

The brittleness of piezoceramics is a bottleneck that limits their further applications in wearable nanogenerators. Therefore, various methods have been adopted to obtain flexible piezoelectric composites [40]. For example, dispersing piezoelectric particles fillers into a polymer matrix to prepare composite films by spin coating [41, 42] or electrospinning [43, 44] methods. However, the content of piezoelectric particles is limited in case the composite films are extraordinary thin and flexible. In addition, the sensitivities of sensors fabricated by the aforementioned methods are unsatisfactory owing to the discontinuous piezoelectric phase structure. To reach a goal of high performance, synthesizing piezoelectric thin films on flexible substrates [45, 46] is regarded as a reliable and alternative tactic. Among numerous flexible substrates, the low-cost glass fiber fabrics with outstanding flexibility and durability are particularly suitable for wearable piezoelectric nanogenerators [45].

In this letter, continuous and well-crystallized BTS thin films were grown on the glass fiber fabric to obtain an ultra-thin (~ 22 μm), superflexible and foldable BTS-GFF/PVDF piezoelectric composite films by simple dipping and spin coating methods. Self-powered sensors based on BTS-GFF/PVDF composite films are utilized for human motion sensing with the thickness of ~ 110 μm and weight of ~ 0.5 g. In the low force region (1 ~ 9 N), BTS-GFF/PVDF sensors have the outstanding performance with voltage sensitivity of ~ 1.23 V N^−1^ and current sensitivity of ~ 41.0 nA N^−1^, which is superior to most previously reported values of piezoelectric sensors and is comparable to those of triboelectric sensors. Moreover, weak disturbances can be detected by the sensors, for instance the mimic motions of human body and the tiny force of falling water drops. This work paves a way for the fabrication of portable and highly sensitive piezoelectric sensors.

## Experimental Section

### Fabrication of BTS-GFF Composites

BTS solution (0.3 mol L^−1^) was prepared by solgel method; tin chloride pentahydrate and titanium butoxide with a molar ratio of 12:88 were dissolved in the mixed solution of ethylene glycol methyl ether and acetylacetone; barium acetate was stirred and dissolved in hot acetic acid at 80 °C. After the barium acetate solution was naturally cooled to room temperature, the two solutions were mixed and stirred evenly, and a small amount of deionized water was added to obtain a bright yellow BTS solution. 0.3 mol L^−1^ BaTiO_3_ (BTO) solution was also prepared by solgel method. The materials used in this work are presented in the S1 section in Supporting Information.

BTS glass fiber fabric composites (BTS-GFF) were prepared by dipping method, respectively; the glass fiber fabrics with high temperature resistance and high flexibility were impregnated in BTS solution and then heated at 120 °C for 5 min and at 450 °C for 10 min. Repeat the dipping process for 14 times and then put it into a muffle furnace and anneal at 800 °C for one hour. BTO-GFF were prepared by the same process for comparison.

### Fabrication of BTS-GFF/PVDF Composite Films

BTS-GFF/PVDF composite films were prepared by spin coating method; 1.5 g PVDF powder was dissolved in 15 mL N, N-dimethylformamide solution, heated and stirred at 80 °C for 7 h to obtain the transparent and viscous PVDF solution. Both sides of the BST-GFF composites were spin-coated with a layer of PVDF, then cured on a hot plate at 120 °C for 10 min, and annealed at 135 °C for 4 h. After cooling to room temperature, the composite films peeled off from the glass sheets. As shown in Figs. S1 and S2a, a superflexible, ultra-thin (thickness of 22 μm), foldable white BTS-GFF/PVDF composite film was prepared. BTO-GFF/PVDF and untreated GFF/PVDF composite films were also prepared by the same process.

### Fabrication of BTS-GFF/PVDF Sensors

A layer of 2 × 3 cm^2^ (see Fig. S2d, e) silver electrode was deposited on both sides of BTS-GFF/PVDF films by magnetron sputtering. The thickness of the sample was 33 μm (Fig. S2b). Copper tape was pasted on both sides to facilitate the detection of electrical signals, and two electrodes were pasted with biaxially oriented polypropylene (BOPP) tape for protection. As shown in Fig. S2c, f, ultra-thin (~ 110 μm) and lightweight (~ 0.5 g) BTS-GFF/PVDF flexible piezoelectric sensors have been fabricated. The blank sample GFF/PVDF sensors and BTO-GFF/PVDF sensors were prepared by the same process for comparison.

### Characterization

The morphology of BTS-GFF/PVDF films were observed under a field emission scanning electron microscope (SEM); X-ray energy-dispersive spectroscopy (EDS) was used to analyze the surface distribution of elements. The crystal structures of the samples were characterized using an X-ray diffractometer (XRD). The curie temperature of BaTi_0.88_Sn_0.12_O_3_ nanopowders was determined by using DSC (differential scanning calorimetry). The dielectric constant (ε_r_) of composite films was tested by Keysight 4990A. In order to obtain the output performance of the prepared devices, the open-circuit voltage (*V*_oc_) and short-circuit current (*I*_sc_) were measured by using Keithley 6514 digital source meter. Different force was applied by linear motor test system to evaluate sensitivity of the sensors.

## Results and Discussion

### Structure and Morphology Analysis

**Fig. 1:**
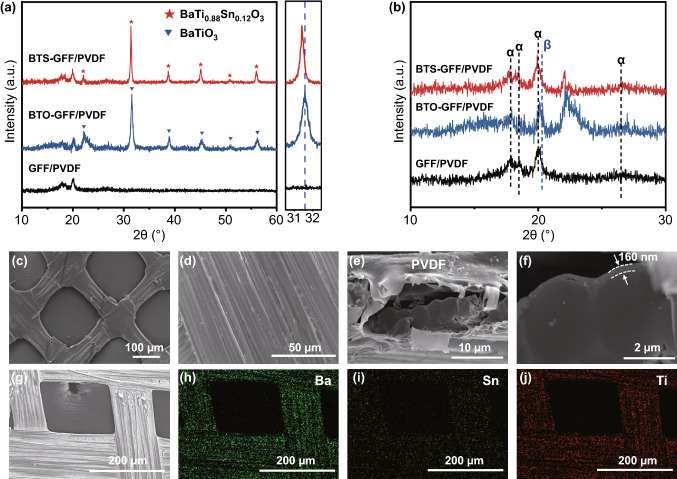
**a, b** XRD patterns of BTS-GFF/PVDF, BTO-GFF/PVDF and GFF/PVDF composite films. **c–f** SEM images of BTS-GFF/PVDF composite film. **g–j** The element mapping result of BTS-GFF composite

The XRD patterns of BTS-GFF/PVDF, BTO-GFF/PVDF, and GFF/PVDF composites as shown in Fig. 1a confirmed the formation of polycrystalline perovskite structure of BTS and BTO films without impurity phase. This result reveals that BTS and BTO films with good crystallinity were successfully prepared by a simple dipping method. Due to the ionic radius of *R*(Ti^4+^) < *R*(Sn^4+^), the XRD peaks of BTS (31.4°) shifted to a lower diffraction than that of BTO (31.58°), which proves the lattice expansion. Figure 1b shows a partial magnification of the diffraction peak of the three composites. Strong diffraction peaks at 2*θ* = 17.7°, 18.4°, 19.9°, and 26.5° correspond to the α crystallographic reflections: (100), (020), (110), and (021) [47], and the weak peak at 20.3° is correspond to the (110) and (120) plane of β-phase [48], indicating the dominance of α-phase in PVDF.

The surface and cross-section morphology of BTS-GFF/PVDF are shown in Fig. 1c–f. The glass fiber fabric has an interconnected structure similar to that of a fishing net, on which the BTS film with a thickness of ~ 160 nm was deposited (Fig. 1f), and the surface of the BTS-GFF composite was covered with a layer of PVDF film (Fig. 1c–f), which benefits the composite film more flexible and sturdier. The elemental composition of BTS-GFF fabric was analyzed by EDS mapping. As schematized in Fig. 1g–j, the elements of Ba, Sn, and Ti are uniformly distributed, indicating that the BTS film grew uniformly on the glass fiber fabric to form an interconnected structure, which is conducive to the transfer of applied force and is necessary for high output [50].

### Sensing Features and Endurance Test

Generally, the human daily motions are in a low frequency range, in order to study the force sensitivity of BTS-GFF/PVDF sensors, the relationship between output signals of the sensors and force were measured under periodic applied force with the low frequency of 0.5 Hz. The open-circuit voltage (*V*_oc_) and short-circuit current (*I*_sc_) of BTS-GFF/PVDF sensors under different applied force (*F*) are shown in Table S1. When the applied force *F* = 1 N, the *V*_oc_ and *I*_sc_ generated by the sensors are 12.2 V and 137 nA, respectively. And a response time of 14 ms and a recovery time of 62 ms have been calculated from the enlarged current versus time curve under the force of 1 N (S3 section). When *F* increased to 55 N, the *V*_oc_ of the sensors reaches to 26.9 V and the corresponding *I*_sc_ reaches to 597 nA.

**Fig. 2:**
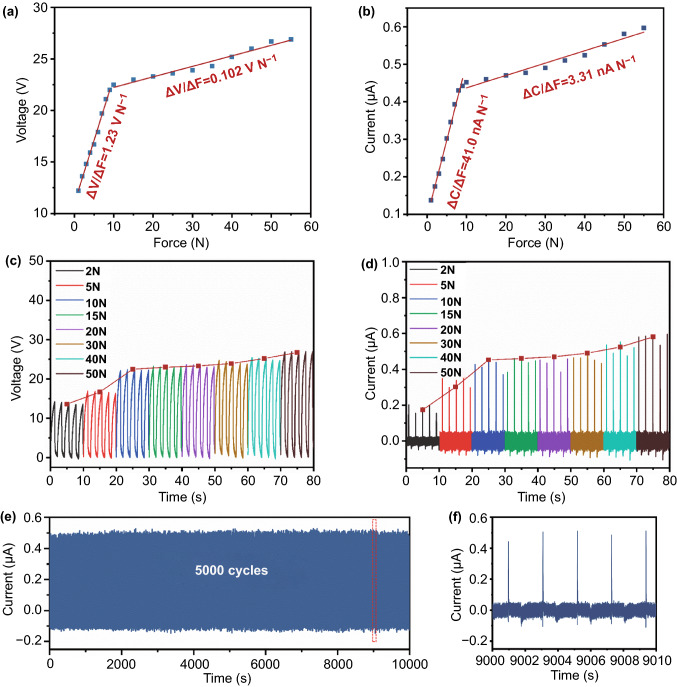
Output performance and stability of BTS-GFF/PVDF sensors. **a** Voltage sensitivity of the BTS-GFF/PVDF sensors. **b** Current sensitivity of the BTS-GFF/PVDF sensors. **c** *V*_oc_ and d *I*_sc_ of BTS-GFF/PVDF sensors with different applied force (2, 5, 10, 15, 20, 30, 40, and 50 N, 0.5 Hz). **e, f** *I*_sc_ of BTS-GFF/PVDF sensors under 5000 pressing cycles (force of 40 N, 0.5 Hz)

As shown in Fig. 2a, b, it can be seen that the output performance of the sensors increased rapidly with the increase of the applied force when the external force *F* was in a low range (1 –9 N). When the applied force *F* is larger than 9 N, the increase of *V*_oc_ and *I*_sc_ slowed down. The sensitivity of BTS-GFF/PVDF sensors, *S*, are calculated according to the formula $S=\Delta V/\Delta F\mathrm{and} \Delta I/\Delta F$, where Δ*F* is the increment of force, Δ*V* and Δ*I* are the voltage and current increments after applying a given force, respectively. When the applied force 1 N < *F* < 9 N, the voltage sensitivity can reach to 1.23 V N^−1^ and the current sensitivity of the sensors can reach to 41.0 nA N^−1^. This result is superior to the sensors prepared by PVDF [49], P (VDF-TrFE) [32], BZT-BCT/P (VDF-TrFE) composite film [50] and is comparable to the triboelectric nanogenerators [2, 51]. When the applied force 10 N < *F* < 55 N, the sensitivity of the sensors decreases to 0.102 V N^−1^ and 3.31 nA N^−1^, respectively. Table 1 shows the sensitivity comparison between this work and other flexible piezoelectric sensors. These results indicate that the piezoelectric response of BTS materials makes it more sensitive to low force (1–9 N). Under the force cycles of 0.5 Hz and 40 N, the reliability and stability of BTS-GFF/PVDF sensors were measured. As shown in Fig. 2e, f, the *I*_sc_ of the sensors remains constant under 5000 force cycles, indicating that the BTS-GFF/PVDF sensors have good reliability.

**Table 1:** Sensitivity comparison between BTS-GFF/PVDF sensors and other flexible piezoelectric sensors

Functional materials	Process	Voltage sensitivity and range of force	References
BTS-GFF/PVDF	Dipping and spin coating	1.23 V N^−1^ (1–9 N) 0.102 V N^−1^ (10–55 N)	This work
Pt-PVDF NFs	Electrospinning	50.8 mV N^−1^ (~ 10 N) 600 mV N^−1^ (10–20 N) 570 mV N^−1^ (20–50 N)	[64]
P(VDF-TrFE) films	Spin coating	50.8 mV N^−1^ (4–80 N)	[65]
P(VDF-TrFE) micropillar array	Patterned EHD pulling	269.4 mV N^−1^ (4–80 N)	[65]
P(VDF-TrFE) nanowires	nanotemplate-based electricity-grown method	458.2 mV N^−1^ (0.1–4 N)	[66]
P(VDF‐TrFE)/BaTiO_3_ nanocomposite micropillars	Nanoimprinting	257.9 mV N^−1^ (5–60 N)	[21]
BZT–BCT/P(VDF–TrFE) composite film	Spin coating	1.11 V N^−1^ (1–10 N)	[67]

**Fig. 3:**
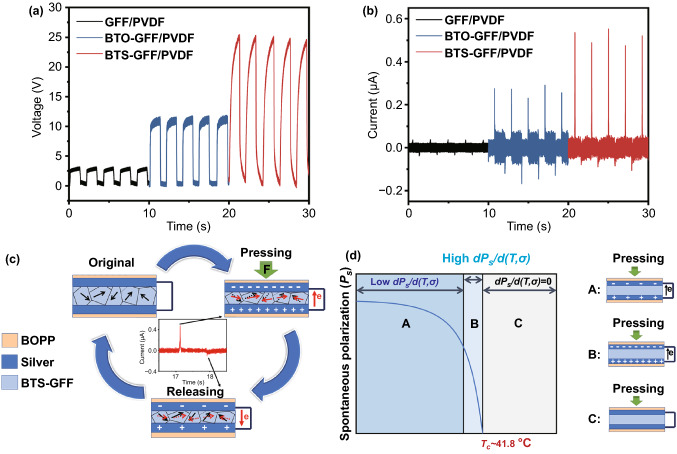
**a, b** Output of GFF/PVDF sensors, BTO-GFF/PVDF sensors, and BTS-GFF/PVDF sensors under the applied force of 40 N, 0.5 Hz. **c** Working mechanism of BTS-GFF/PVDF sensors. **d** Effect of *T*_c_ on polarization [39] and electric responses of sensors under different working temperature

In order to verify that the output performance of BTS-GFF/PVDF sensors is attributed to the excellent piezoelectric performance of BTS near room temperature, the output of the blank GFF/PVDF, BTO-GFF/PVDF, and BTS-GFF/PVDF sensors was tested under the external periodic force of 40 N and 0.5 Hz (Fig. 3a, b). The blank sample has an open-circuit voltage (*V*_oc_) of ~ 3 V and a short-circuit current (*I*_sc_) of ~ 30 nA [52–54] which should be attributed to the small amount of piezoelectric β-phase of PVDF in samples. Under the same applied force, the *V*_oc_ and *I*_sc_ of BTO-GFF/PVDF sensors are 11.8 V and 272 nA, respectively. The output values of BTS-GFF/PVDF sensors increase significantly and can reach to ~ 25 V and ~ 524 nA, respectively, which demonstrates that the high output of BTS-GFF/PVDF sensors is mainly owing to the piezoelectric effect of BTS. Besides, the increase of dielectric constant may also be beneficial to the increase of charge density, so is the output of devices (Fig. S6).

The good performance of the BTS-GFF/PVDF sensors should be attributed to the low energy barrier for triggering polarization rotation near *T*_c_. The *T*_c_ of BaTi_0.88_Sn_0.12_O_3_ nanopowders was determined by using DSC, as shown in Fig. S4, two obvious steps can be seen in the DSC curve, the ending of which is ~ 41.8 °C, indicating the completion of ferroelectric–paraelectric phase transition, and could be regarded as *T*_c_ of BTS nanopowders. The working mechanism of the sensors is schematically described in Fig. 3c. In the absence of external force, the polar nanoregions in the BTS-GFF/PVDF films are arranged randomly and are in a state of equilibrium [34]. Therefore, there is no output of electrical signals. When an external force is applied to the sensor, the polar nanoregions inside the films are deflected to form a built-in electric field, resulting in induced charges and currents [55–57]. When the force is released, the internal electric field disappears, resulting in a smaller reverse current [34]. When the working temperature of the sensor is close to the *T*_c,_ the internal spontaneous polarization of the films is more likely to rotate under the action of external force [39], resulting in more induced charges and larger output signal (Fig. 3d). Therefore, BTS thin film materials with low *T*_c_ are highly desirable for human wearable devices. And this work mechanism also could be used to explain the decreased sensitivity under large force. Figure S3c illustrate the changes of polar nanoregions in BTS under different force. Lattice strain is proved to be able to induce ferroelectric polarization [58–61]. Therefore, strain-induced polarization rotation increases with the increase of mechanical stress (the deflection of black arrows to red arrows). However, the ferroelectric polarization will approach to saturation (the deflection of black arrows to green arrows). Thus, a lower sensitivity is found in the BTS-GFF/PVDF sensors when the external force reaches to a critical value.

### Applications

**Fig. 4:**
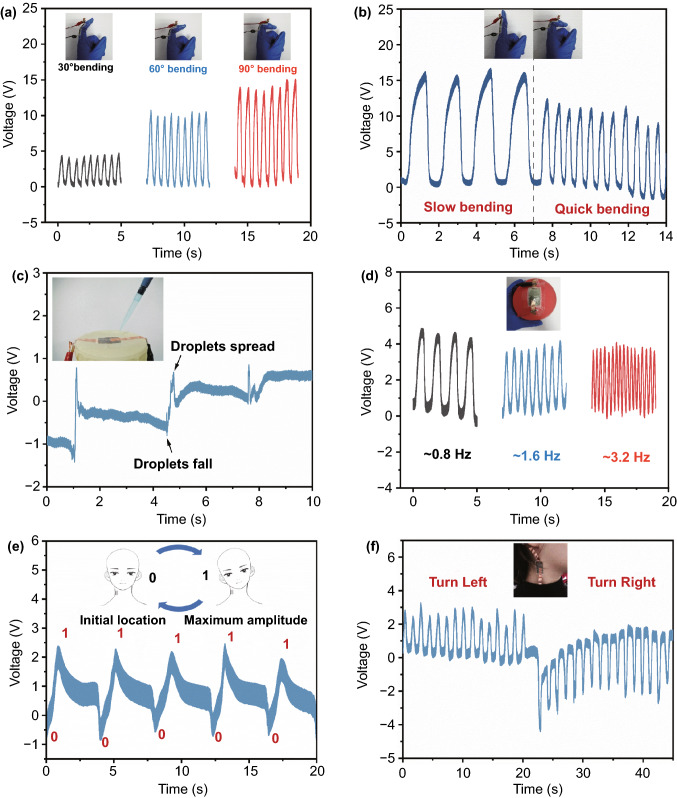
Output voltage of BTS-GFF/PVDF sensors when the finger is bent at **a** different angle (30°, 60°, 90°) and **b** different speeds. **c** Voltage signal generated by BST-GFF/PVDF sensor under falling water droplets. The output voltage of BTS-GFF/PVDF sensors when **d** pressing the balloon at different frequencies (~ 0.8 Hz, ~ 1.6 Hz, ~ 3.2 Hz), **e** the tester deflects her head to the left at a continuous frequency of 0.25 Hz and **f** the tester turns her head left and right at the frequency of 0.75 Hz

The BTS-GFF/PVDF sensors were used to detect the movements of the human body. We attached the sensor to the finger to detect the motion amplitude and frequency of the human finger (Movie S1). As shown in Fig. 4a, the BTS-GFF/PVDF sensors can stably output *V*_oc_ of ~ 4.4 V when the finger is bent 30°. When the bending angle of the finger increases to 60° and 90°, the *V*_oc_ can reach to ~ 10.3 and ~ 14.1 V, respectively. The results may be attributed to the larger contact area and force between finger and BTS-GFF/PVDF sensor when the bending angle increased. When bending fingers at different speeds, BTS-GFF/PVDF sensors can also output voltages at different frequencies, the output signal is shown in Fig. 4b. A high bending frequency led to a low output performance, which should be attributed to the larger force acting on the sensor from the joint during the slow bending process. As shown in the Movie S1, when the tester bends the finger with different frequency, the contact time between the finger and the sensors will be different. The force acting on the sensors only could reach up to saturation when the bending frequency is small enough. Thus, the output performance will decrease as the bending frequency increases. The results of this work indicate that BTS-GFF/PVDF sensors can be used to monitor the frequency and amplitude of slow human motion, and can be used in the medical field of human rehabilitation motion monitoring and intelligent robot motion monitoring in the future [1].

In order to verify the detection performance of the sensor with weak force in practical application, we designed an experiment to detect the tiny forces of raindrops (Fig. 4c). When the water droplets (~ 500 mg) fall on the sensor from the height of ~ 3 cm, BTS-GFF/PVDF sensor generates an obvious voltage signal (*V*_oc_ ≈ 1.6 V) while there is almost no signal generated by GFF/PVDF sensor (Fig. S5), which proves that the output of BTS-GFF/PVDF sensor is mainly due to the piezoelectric effect of BTS. This result shows that BTS-GFF/PVDF sensor can detect very small mechanical forces. Considering the superiority of the sensors in detecting tiny force, we attached BTS-GFF/PVDF sensor on the surface of the balloon so as to mimick the fluctuation of the abdomen while breathing, the heartbeats and so on, the deformations produced on the balloon surface by regular interval pressing (at frequency of ~ 0.8, ~ 1.6, and ~ 3.2 Hz) can be detected (Fig. 4d and Movie S2). As the frequency increased from 0.8 to 3.2 Hz, the *V*_oc_ decreases from ~ 4.6 to ~ 3.7 V, which is consistent with the variation of the output voltage signal with the finger bending frequency mentioned above. When the speed of pressing the ball slows down, the contact between the sensor and the surface of the balloon is more sufficient, and the output signal is larger. In the process of pressing the balloon, the change of the motion amplitude of the balloon surface is not as large as that of the finger bending, therefore the *V*_oc_ of the sensor to detect the balloon motion deformation is smaller than that of the finger bending, and the change of *V*_oc_ with the pressing frequency is less obvious than that of the finger bending. However, the BTS-GFF/PVDF sensors are still very sensitive to the balloon motion frequency. This work shows that BTS-GFF/PVDF sensors can be used to detect small movements, and in the medical field, it can be used to count and monitor the fluctuation of human abdomen or heartbeats in the future [4, 62].

We also attached BTS-GFF/PVDF sensor on the left side of a person’s head and neck skin to monitor the movements. In order to mimick this head movements during fatigue driving, the tester deflected the head to the left at a continuous frequency of 0.25 Hz. At the undeflected state of the head, the position is recorded as point 0 and at the maximum deflection to the left, the position is recorded as point 1. In case the tester deflects the head from position 0 to position 1, the output signal of the sensors is about + 3 V (Fig. 4e). On the contrary, if the tester’s head returns from the maximum deflection position 1 to the initial position 0, the output voltage decreases to the initial value. When the tester turns her head to the left or right at a frequency of 0.75 Hz, the output voltage of BTS-GFF/PVDF sensors is about + 3 and − 3 V, respectively. When the tester changes the direction of the twist, there is an obvious change in the output voltage of the sensor, as shown in Fig. 4f. This head movements experiment shows that BTS-GFF/PVDF sensors can be used to monitor human head movements and detect fatigue driving [63]. In the future, the sensors may also be used to remind the neck movements of sedentary people in office.

## Conclusions

In summary, we have developed a method to fabricate superflexible BTS-GFF/PVDF composite films and highly sensitive sensors. It is found that BTS with low *T*_c_ is a benefit for flexible piezoelectric sensors because small alterations of force will trigger large changes in polarization. The voltage sensitivity and current sensitivity of the as prepared sensors can reach to 1.23 V N^−1^ and 41.0 nA N^−1^ as the external force increase from 1 to 9 N. Under the action of 5000 cycles of 40 N external force, the output of the sensors shows little change, which proves that the sensors have very good reliability. We also demonstrated that BTS-GFF/PVDF sensors can detect very tiny forces (waterdrops falling) and human motions (finger bending, head turning and mimic motions by pressing the balloon), indicating this kind of sensor has a very wide application prospect in medical treatment, motion monitoring and artificial intelligence robots in the future.

## Supplementary Information

Below is the link to the electronic supplementary material.

Supplementary file1 (PDF 512 kb)

Supplementary file2 (MP4 4520 kb)

Supplementary file3 (MP4 2981 kb)
