# Supplementary material for: Superflexible and Lead-Free Piezoelectric Nanogenerator as a Highly Sensitive Self-Powered Sensor for Human Motion Monitoring
Source: Nanomicro Lett. 2021 Apr 30;13:117. doi: 10.1007/s40820-021-00649-9 (PMC8093345; doi:10.1007/s40820-021-00649-9)
Supplement: Supplementary file 1 — Supplementary file1 (PDF 512 kb) [file 40820_2021_649_MOESM1_ESM.pdf]

Supporting Information for

Superflexible and Lead-Free Piezoelectric Nanogenerator as a Highly Sensitive Self-Powered Sensor for Human Motion Monitoring

Di Yu[1], Zhipeng Zheng[1], Jiadong Liu[1], Hongyuan Xiao[1], Geng Huangfu[1], Yiping Guo[1, *]

[1]State Key Laboratory of Metal Matrix Composites, School of Materials Science and Engineering, Shanghai Jiao Tong University, Shanghai 200240, P. R. China

*Corresponding author. E-mail: ypguo@sjtu.edu.cn (Yiping Guo)

S1 Materials

Barium acetate ($C_4H_6O_4.Ba$, MACKLIN), Tin chloride pentahydrate ($SnCl_4·5H_2O$, aladdin), Acetylacetone ($C_5H_8O_2$, aladdin)**,** Acetic Acid Glacial (CH_3CO_2H, GENETAL-REAGENT), Titanium butoxide ($C_{16}H_{36}O_4Ti$, aladdin), 2-Methoxyethanol ($C_3H_8O_2$, aladdin). PVDF powders (Kynar® 900, $M_w \approx 1000000$, Arkema Inc), N, N-Dimethylformamide (DMF, aladdin).

S2 Flexibility, Thickness and Weight of Samples

The flexibility of BTS-GFF/PVDF composite film is shown in Fig. S1. The film can be bent at will and can be restored to its original state under any folding. The thickness of the prepared sample was measured by spiral micrometer. The thickness of BTS-GFF/PVDF composite film is about 22 μm. After plating a layer of 2 cm × 3 cm silver electrode on both sides, the thickness increases to ~33 μm. Finally packaged with BOPP tape, the thickness of BTS-GFF/PVDF sensor is ~110 μm. The sensor is very light, with a weight of only 0.5002 g.

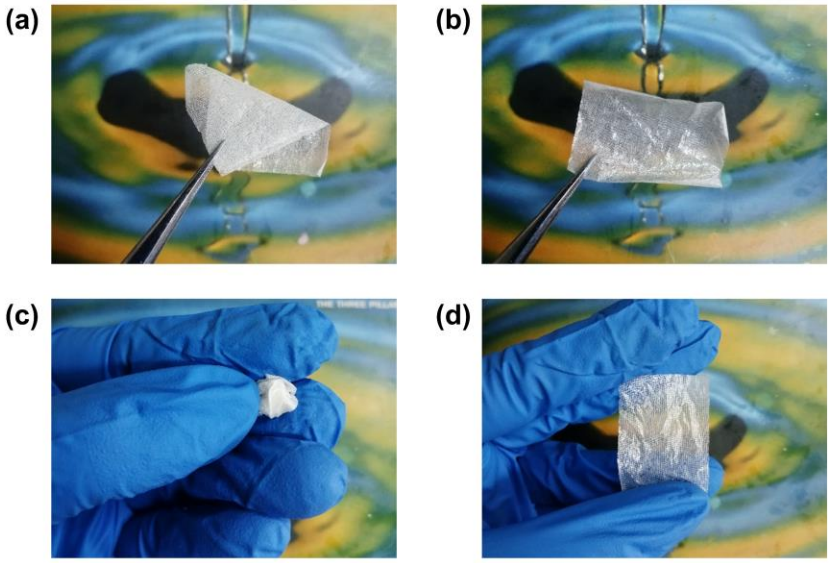

Fig. S1 Flexibility of BTS-GFF/PVDF composite film: (**a**) folding, (**b**) unfolding, (**c**) kneading, and (**d**) unfolding

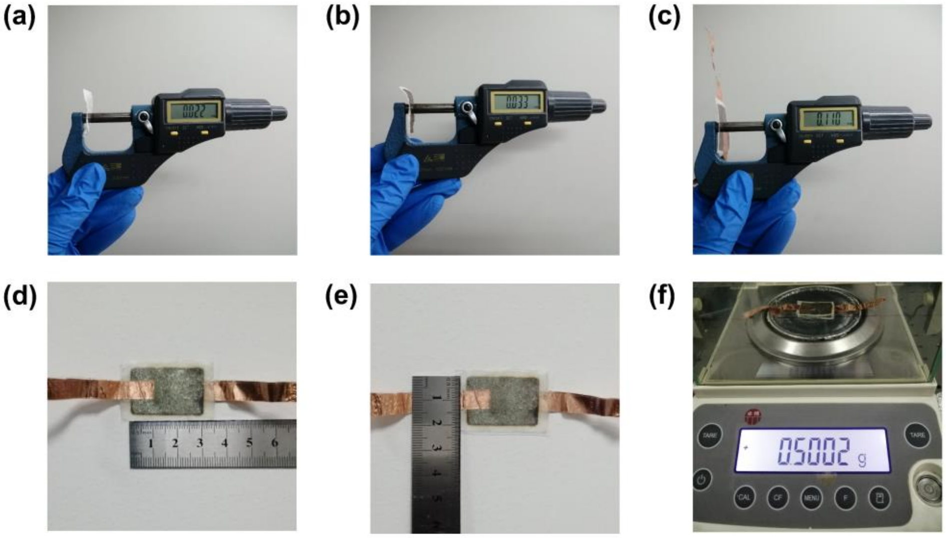

Fig. S2 Thickness and weight of samples. (**a**) Thickness of BTS-GFF/PVDF composite film. (**b**) Thickness of BTS-GFF/PVDF composite film with silver-plated electrodes on both sides. (**c**) Thickness of BTS-GFF/PVDF sensor. (**d, e**) The electrode area of BTS-GFF/PVDF sensor. (**f**) The weight of BTS-GFF/PVDF sensor

S3 Output, Sensing Time and Working Mechanism of BTS-GFF/PVDF Sensors

The linear motor system was used to apply different external pressure to BTS-GFF/PVDF sensor. The open-circuit voltage (V_{oc}) and short-circuit current (I_{sc}) of the sensor were measured by Keithley 6514 digital source meter, as shown in Table S1. The measuring range of the current testing software is 200 nA and 2 μA. When the external force is small (1N~4N), we choose 200 nA as the measuring range; When the external force increases (≥ 5N), the output current increases, so we choose 2 μA as the measuring range, the corresponding base noise becomes larger (Fig. S3a). There is obvious base noise in the current figures, so we calculate the limit of detection according to the current curve. The limit of detection is three times the height of base noise peak. When the measuring range is 200 nA, the average peak value of base noise is about ±0.0223 μA, and the limit of detection is ±0.066 μA. When the measuring range is 2 μA, the average peak value of base noise is ±0.044 μA, and the limit of detection is ±0.132 μA. A response time of 14 ms and a recovery time of 62 ms have been calculated from the enlarged current versus time curve under the force of 1 N, 0.5 Hz. We also illustrate the changes of polar nano regions in BTS under different force according to the working mechanism to explain that the sensitivity will become small when the force reaches to a critical value.

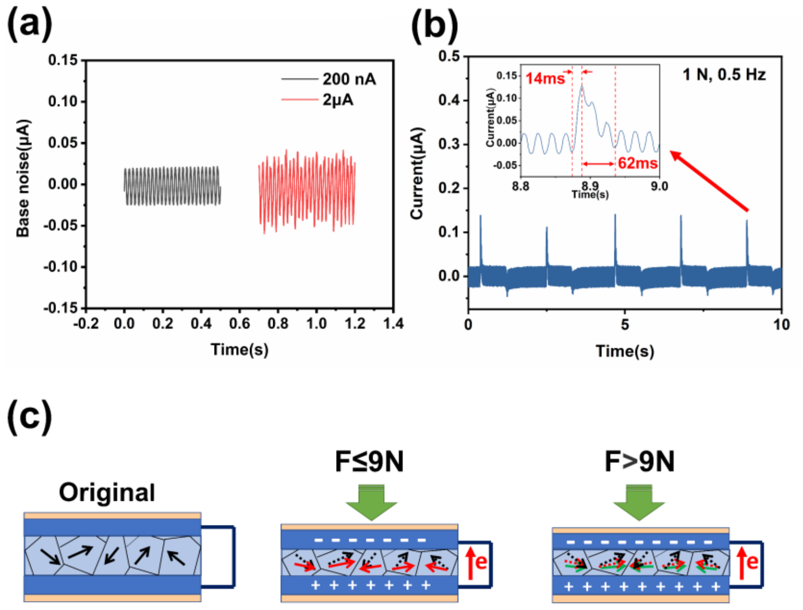

Fig. S3 (**a**) Current base noise of Keithley 6514 digital source meter under different measuring range (200 nA and 2 μA). (**b**) The sensing response time and recovery time of BTS-GFF/PVDF sensors. (**c**) The working mechanism of BTS-GFF/PVDF sensors under different force

Table S1 V_{oc} and I_{sc} of BTS-GFF/PVDF sensor under different pressures

**Pressure (N)**	V_{oc}**(V)**	I_{sc}**(μA)**
1	12.2	0.137
2	13.6	0.174
3	14.8	0.208
4	15.9	0.247
5	16.7	0.302
6	17.9	0.346
7	19.7	0.393
8	21.1	0.430
9	22.0	0.442
10	22.5	0.452
15	23.0	0.460
20	23.3	0.470
25	23.6	0.477
30	23.9	0.490
35	24.3	0.510
40	25.2	0.524
45	26.0	0.553
50	26.7	0.581
55	26.9	0.597

S4 Curie Temperature of $BaTi_{0.88}Sn_{0.12}O_3$ Materials

$BaTi_{0.88}Sn_{0.12}O_3$ nano powders were prepared by sol-gel method. DSC test was reported to determine the characteristic temperatures of phase transition processes. As shown in Fig. S4, two obvious steps can be seen in the DSC curve, the ending of which is ~41.8 °C, indicating the completement of ferroelectric-paraelectric phase transition, and could be regarded as T_c of BTS nano powders.

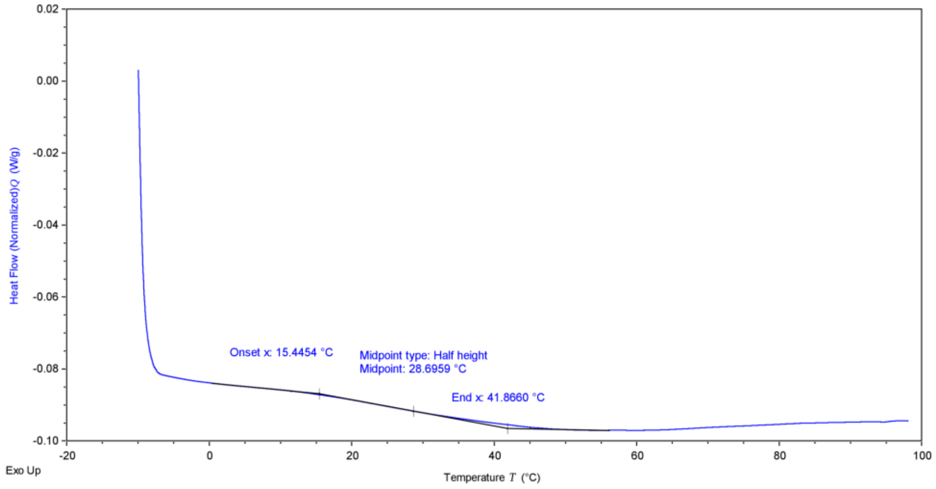

Fig. S4 DSC curve of $BaTi_{0.88}Sn_{0.12}O_3$ nano powders

S5 Voltage Signal of GFF/PVDF Sensors

In order to prove that piezoelectric effect plays a dominant role in the progress of droplets fall and spread, we tested the output performance of GFF/PVDF sensors under the same experimental conditions. The result is presented in Fig. S5. There is almost no signal generated by the sensor and the output of BTS-GFF/PVDF sensor is mainly due to the piezoelectric effect of BTS.

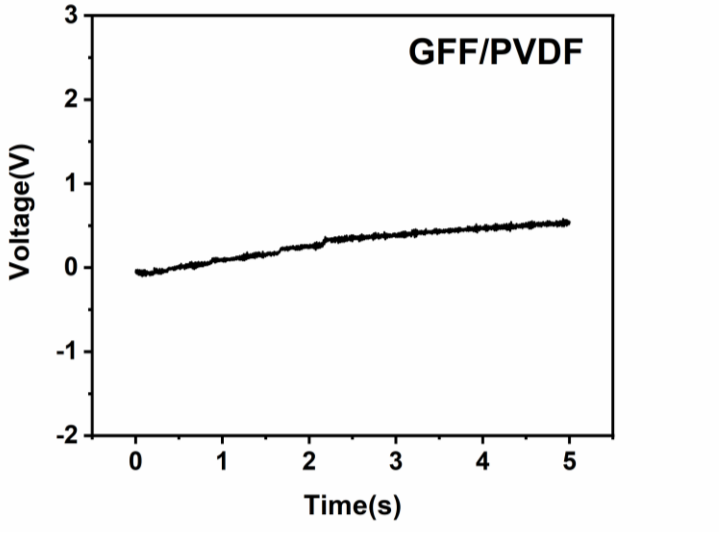

Fig. S5 Voltage signal of GFF/PVDF sensors under falling water droplets

S6 Dielectric Properties of Composite Films

The dielectric constant (ε_r) of BTS-GFF/PVDF composite ($\varepsilon_r \approx 56.8$) is twice that of GFF/PVDF ($\varepsilon_r \approx 27.4$). The increase of dielectric constant is beneficial to the increase of charge density, which will enhance the output of devices.

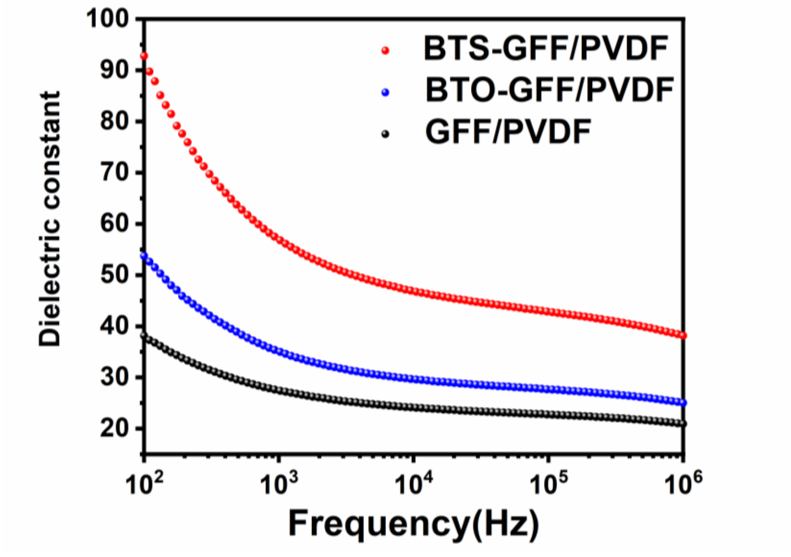

Fig. S6 Dielectric constant (ε_r) of BTS-GFF/PVDF, BTO-GFF/PVDF and GFF/PVDF composite films

Supplementary Movies

Movie S1 Open-circuit voltage (V_{oc}) generation under finger bending.

Movie S2 Open-circuit voltage (V_{oc}) generation under balloon pressing.
